# Family History of Premature Coronary Artery Disease (P-CAD)—A Non-Modifiable Risk Factor? Dietary Patterns of Young Healthy Offspring of P-CAD Patients: A Case-Control Study (MAGNETIC Project)

**DOI:** 10.3390/nu10101488

**Published:** 2018-10-12

**Authors:** Tadeusz Osadnik, Natalia Pawlas, Marta Lonnie, Kamila Osadnik, Mateusz Lejawa, Lidia Wądołowska, Kamil Bujak, Martyna Fronczek, Rafał Reguła, Marcin Gawlita, Joanna Katarzyna Strzelczyk, Marta Góral, Marek Gierlotka, Lech Poloński, Mariusz Gąsior

**Affiliations:** 1 2nd Department of Cardiology and Angiology, Silesian Center for Heart Diseases, Marii Skłodowskiej-Curie 9, 41-800 Zabrze, Poland; 2 Department of Pharmacology, School of Medicine with the Division of Dentistry in Zabrze, Jordana 38, 41-808 Zabrze, Medical University of Silesia, 40-055 Katowice, Poland; n-pawlas@wp.pl (N.P.); kamila.osadnik@onet.pl (K.O.); mateusz.lejawa@gmail.com (M.L.); 3 Institute of Occupational Medicine and Environmental Health, Kościelna 13, 40-001 Sosnowiec, Poland; 4 Department of Human Nutrition, Faculty of Food Science, University of Warmia and Mazury in Olsztyn, Sloneczna 45f, 10-718 Olsztyn, Poland; marta.lonnie@uwm.edu.pl (M.L.); lidia.wadolowska@uwm.edu.pl (L.W.); 5 Genomics Laboratory, Kardio-Med Silesia Science and Technology Park, Marii Skłodowskiej-Curie 10C, 41-800 Zabrze, Poland; 6 3rd Department of Cardiology, School of Medicine with the Division of Dentistry in Zabrze, Medical University of Silesia in Katowice, Silesian Center for Heart Diseases, Marii Skłodowskiej-Curie 9, 41-800 Zabrze, Poland; kamil_bujak@o2.pl (K.B.); r.regula@gmail.com (R.R.); m.gierlotka@sccs.pl (M.G.); scchs@sum.edu.pl (L.P.); m.gasior@op.pl (M.G.); 7 Department of Medical and Molecular Biology, School of Medicine with the Division of Dentistry in Zabrze, Jordana 19, 41-808 Zabrze, Medical University of Silesia, 40-055 Katowice, Poland; martynafronczekk@vp.pl (M.F.); jstrzelczyk@sum.edu.pl (J.K.S.); 8 Department of Environmental Medicine and Epidemiology, School of Medicine with the Division of Dentistry in Zabrze, Jordana 19, 41-808 Zabrze, Medical University of Silesia, 40-055 Katowice, Poland; marcin.gawlita@wp.pl; 9 Students’ Scientific Society, 3rd Department of Cardiology, School of Medicine with the Division of Dentistry in Zabrze, Medical University of Silesia in Katowice, Silesian Center for Heart Diseases, Marii Skłodowskiej-Curie 9, 41-800 Zabrze, Poland; martagoral5@wp.pl; 10 Department of Cardiology, University Hospital in Opole, Faculty of Natural Sciences and Technology, Institute of Medicine, University of Opole, W. Witosa 26, 45-401 Opole, Poland

**Keywords:** dietary patterns, family history, FFQ-6, PCA, premature coronary artery disease, P-CAD

## Abstract

Dietary habits of healthy offspring with a positive family history of premature coronary artery disease (P-CAD) have not been studied so far. The aim of this study was twofold: (1) to identify dietary patterns in a sample of young healthy adults with (cases) and without (controls) family history of P-CAD, and (2) to study the association between dietary patterns and family history of P-CAD. The data came from the MAGNETIC case-control study. The participants were healthy adults aged 18–35 years old, with (*n* = 351) and without a family history of P-CAD (*n* = 338). Dietary data were collected with food frequency questionnaire FFQ-6. Dietary patterns (DP) were derived using principal component analysis (PCA). The associations between the adherence to DPs and family history of P-CAD were investigated using logistic regression. Two models were created: crude and adjusted for age, sex, smoking status, place of residence, financial situation, education, and physical activity at leisure time. Three DPs were identified: ‘prudent’, ‘westernized traditional’ and ‘dairy, breakfast cereals, and treats’. In both crude and adjusted models, subjects with family history of P-CAD showed higher adherence by 31% and 25% to ‘westernized traditional’ DP (odds ratio (OR) 1.31, 95% confidence interval (95% CI): 1.12–1.53; *p* < 0.005; per 1 unit of standard deviation (SD) of DP score and _adj_OR 1.25, 95% CI: 1.06–1.48; *p* = 0.007; per 1 unit of SD of DP score, respectively). Young healthy adults with family history of P-CAD present unfavorable dietary patterns and are potentially a target group for CAD primary prevention programs.

## 1. Introduction

Premature coronary artery disease (P-CAD) has a multifactorial etiology and is most likely a mixture of genetic and environmental factors. The relationship between family history of P-CAD in first-degree relatives and increased risk of atherosclerosis-based diseases is well described [1,2,3,4]. A particularly robust factor affecting the offspring is myocardial infarction (MI) occurrence before the age of 55 for men and 65 for women [2]. It has been estimated that the age-adjusted odds ratio of cardiovascular events is around 2.5 greater among individuals with family history of P-CAD [1], with a further 10-fold increase if the first-degree relative was affected under the age of 45 [3]. Among patients with family history of P-CAD, a more frequent occurrence of hypertension, hypercholesterolemia, abdominal obesity, and smoking has been reported [4]. A better understanding of the genetic and environmental background of the disease could result in a substantial progress in successful screening [5]. However, this also resulted in the focus of care provision being diverted from primary care to early diagnosis and pharmacological treatment. As highlighted by Jeemon et al. [6], a shift from a ‘reactive’ to a ‘proactive’ approach in the preventative management of patients with family history of P-CAD should receive more support.

Prevention mostly relies on lifestyle modifications. Poor dietary habits are one of the critical environmental factors for CAD occurrence in general population [7]. Conversely, healthy dietary patterns—characterized by high intake of low-fat dairy products, whole grains, vegetables, and fruits—are associated with a lower risk of death due to CAD and a lower risk of non-fatal MI incidence within nearly 28 years of observation [8]. Inadequate nutrition and smoking are highly prevalent among cardiovascular patients [9] and may cluster with other unfavorable behaviors such as excessive drinking and low activity level [10], increasing the cumulative effect of multiple risks. A study among young adults with P-CAD revealed that their smoking habit was correlated with parental smoking [11]. The study, however, did not provide information regarding characteristics of the diet in affected families. We hypothesized that, in a similar manner, unhealthy dietary habits may be passed on to the offspring in families with a history of P-CAD.

To our knowledge, dietary habits of the offspring of P-CAD patients have not been yet studied. Particularly, in the context of dietary patterns (DPs) derived with the use of multidimensional statistical techniques. Since no foods nor nutrients are consumed in isolation, the proposed DPs approach appears to better reflect the complexity of ‘real life’ diet, by analyzing the associations between combinations of different foods, specific to the studied population, and health outcomes [12]. Filling this research gap can provide an essential insight into the associations between diet and familial history in this specific group of patients.

The aim of this study was twofold: (1) to identify dietary patterns in a sample of young healthy adults with (cases) and without (controls) family history of P-CAD, and (2) to study the association between dietary patterns and family history of P-CAD.

## 2. Materials and Methods

### 2.1. Study Design and Sample

**Figure 1:**
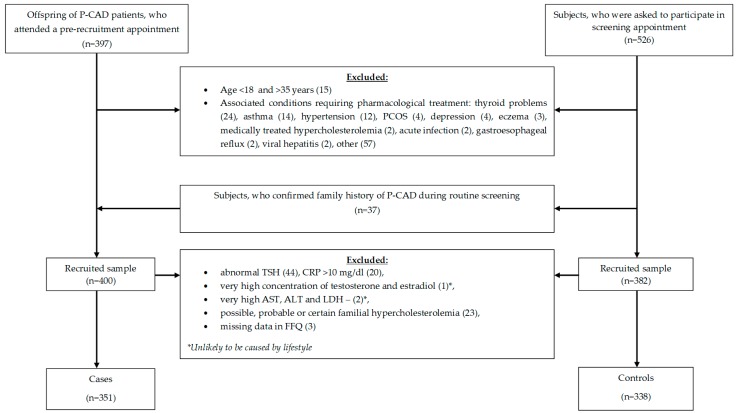
Recruitment flow-chart.

The following analysis is a part of the Metabolic and Genetic Profiling of Young Adults with and without a Family History of Premature Coronary Heart Disease (MAGNETIC) project, which is a case-control study that aims at analyzing classical and genetic risk factors of CAD in healthy young adults with and without a family history of P-CAD. Study design and methodology of the MAGNETIC project have been described previously [13]. In brief, the study sample was recruited between July 2015 and October 2017. The frequency matching method was used; gender as a matching variable. The inclusion criteria were: age ≥18 and ≤35 years old, P-CAD (myocardial infarction, percutaneous coronary intervention or coronary artery bypass grafting before the age of 55 in men, and 65 in women) in first-degree relatives (cases) or no P-CAD in first-degree relatives (control group). The exclusion criteria for both groups were: age <18 or >35 years, failure to provide informed consent, pregnancy, lactation, and acute or chronic diseases requiring pharmacotherapy. Subjects with a positive family history of P-CAD were recruited in two ways: (1) from healthy subjects aged 18–35 years, who were asked to participate in screening appointment at the Silesian Center for Heart Diseases, who provided documented proof of P-CAD history in their first-degree relatives, and (2) from the healthy offspring of the center patients, hospitalized in 2010–2017 due to P-CAD, who gave permission to be contacted once the treatment was completed. The control group was recruited from healthy subjects aged 18–35 years, who were asked to participate in screening appointment at the center, who confirmed no family history of P-CAD. In total, 689 subjects met the inclusion criteria and were included in the study. Of these, 351 (50.9%) had a parent with documented P-CAD (cases), and 338 (49.1%) subjects reported no family history of P-CAD (controls). The recruitment flow chart is presented in Figure 1.

### 2.2. Ethical Approval

The study was conducted following the Declaration of Helsinki and good clinical practice guidelines. The study protocol has been approved by the Ethics Committee at Institute of Occupational Medicine and Environmental Health, Sosnowiec (resolution No. 03/2013). Informed, written consent was obtained from all subjects enrolled to the study.

### 2.3. Dietary Data Collection

Dietary data were collected with a validated 61-item food frequency questionnaire (FFQ-6) for adolescents and adults [14]. The questionnaire covered a whole diet, including a wide variety of foods consumed in Poland. A validation procedure of the questionnaire was carried out in healthy females aged 13–21 years (data not published, paper in preparation). In brief, the internal compatibility of the questionnaire was tested by 97 respondents. The questionnaire was completed by respondents twice (test and retest after two weeks). Fleiss’ Kappa for food items ranged from 0.32 to 0.72 (on average, 0.52), and there were 94% of food items with Fleiss’ Kappa above 0.4 (cut-off for acceptable compatibility for dietary data) [15]. Compatible classification of subjects (into the same category in test and retest) ranged from 51% to 89% (on average, 68%). Therefore, the FFQ-6 proved ‘acceptable’ to ‘very good’ internal compatibility and was considered as an appropriate tool to evaluate dietary habits. The wide scope of FFQ-6 application was confirmed previously by its use in a randomized controlled trial among pediatric coeliac disease patients on a gluten-free diet [16], in the study regarding genetic-specific nutritional intervention in adult patients with non-alcoholic fatty liver disease [17], and in lung and breast cancer patients [18]. 

In this study, the self-administered version of FFQ-6 was used. Trained researchers were handing the questionnaires to the enrolled participants providing guidance and assistance as required, on the one-to-one basis. Questionnaires were completed and returned along with signed informed consents, prior to further data collection. Food frequency consumption covering the past 12 months was collected. Respondents could select one of six categories of frequency consumption, described in a range from ‘never or very rarely’ to ‘few times a day’. For further analysis, the frequency of consumption was recalculated and expressed as times/day as follows: ‘never or very rarely’ = 0; ‘once a month or less’ = 0.025; ‘several times a month’ = 0.1; ‘several times a week’ = 0.571; ‘daily’ = 1; ‘few times a day’ = 2 [14]. Some of the food items were combined by summing their daily frequency consumption (times/day) into 26 food groups (Table 1).

### 2.4. Confounding Factors

Based on the identified in the literature common risk factors of P-CAD, the following confounders were considered: age, sex, smoking status, place of residence, financial situation, level of education and physical activity at leisure time. Data on confounders was obtained with a validated questionnaire (KomPAN) using closed structured questions [19,20]. Physical activity at leisure time was categorized as low (sitting, screen time, reading, light housework, walking less than 2 h a week), moderate (walking, cycling, moderate exercise, working at home or other light physical activity performed 2–3 h/week), or high (cycling, running, working at home or other sports activities requiring physical effort over 3 h/week). Other variables were collected in three or four categories and then classified in dichotomous categories as follows: smoking (non-smoker or past smoker vs. current smoker), place of residence (city <20,000 inhabitants vs. city >20,000 inhabitants), financial situation (below average or average vs. above average, based on respondent’s declaration), and education (primary or secondary vs. higher).

### 2.5. Statistical Analysis

Smoking status and financial situation variables contained missing values for 2 (0.28%) and 1 (0.14%) subjects respectively. Food frequency questionnaire contained 51 missing values out of 42,029 element response matrix (0.1%). Missing values were imputed using the missForest data imputation algorithm [21], separately for clinical characteristics variables and food frequency questionnaire variables. All variables mentioned above, including the outcome variable, were entered into the multiple imputation algorithm; the missForest R–package was used [22]. The frequency of consumption of 26 food groups (times/day) was standardized so that that values had mean of 0 and standard deviation of 1. Principal component analysis (PCA) with varimax rotation, based on the correlation matrix of standardized variables, was used for dietary patterns identification and was described elsewhere in detail [23]. In brief, each component identifies a dietary pattern, that is a linear combination of questionnaire items. Components to retain were based on their interpretability and eigenvalues (>1) and a break-point identified in Scree test. The contribution of each questionnaire item to each dietary pattern is reflected by the item’s factor loading. Rotated factor loadings > |0.30| were considered to be of significant contribution to identified dietary patterns. Dietary patterns were labeled according to variables with highest loadings for each dietary pattern. For each subject, a dietary pattern score that reflects adherence to the dietary pattern was calculated (as a sum of the product of the food frequency consumption and factor loading for 26 food groups). 

Unconditional logistic regression analysis was used to assess the association between adherence to identified dietary patterns and family history of P-CAD [24]. Logistic regression models were adjusted for potential confounders: age, sex, smoking status, place of residence, financial situation, education, physical activity at leisure time (as mentioned in the Section 2.4). Separate models were built for dietary pattern scores as a continuous term (per one unit of standard deviation (SD) of dietary pattern score) and for the tertile intervals calculated for each dietary pattern score. The bottom tertile of each dietary pattern score was treated as a reference. Two-sided Cochrane–Armitage test for proportions was used to assess a trend in the percentage of patients with family history of P-CAD across each dietary pattern score tertile [25,26]. *p*-values < 0.05 were considered to be statistically significant. 

## 3. Results

### 3.1. Sample Characteristics

Compared with controls, patients with family history of P-CAD were older (average age 28.6 (standard deviation (SD) 4.6) vs. 27.3 (SD 4.2) years), more often current smokers (27.4% vs. 17.2%, respectively), and less often described their financial situation as above average (21.1% vs. 29.2%, respectively). No differences between the groups were observed regarding sex, place of residence, level of education, and physical activity at leisure time (Table 2).

### 3.2. Dietary Patterns

**Figure 2:**
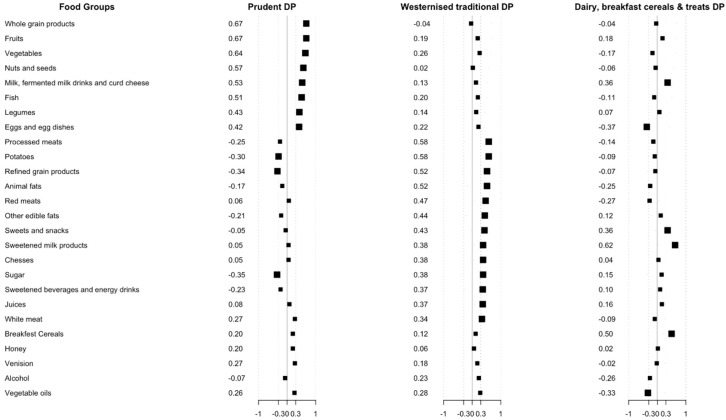
Factors loadings for the three dietary patterns (DPs) identified by principal component analysis. The larger squares represent items with factor loading ≥ |0.3|.

The principal component analysis led to the identification of three dietary patterns explaining 13, 12, and 6% of the variance of consumption in 26 food groups. Total explained variation of these three dietary patterns was 31%. The first dietary pattern—‘prudent’—was characterized by frequent consumption whole grain products (0.67); fruit (0.67); vegetables (0.64); nuts and seeds (0.57); milk, fermented milk drinks, and curd cheese (0.53); fish (0.0.51); legumes (0.43); eggs and egg dishes (0.42); and infrequent consumption of sugar (−0.35) and refined grain products (−0.34). The second dietary pattern, labelled ‘westernized traditional’ was characterized by frequent consumption of processed meats (0.58); potatoes (0.58); refined grain products (0.52); red meats (0.47); other edible fats (0.44); sweets and snacks (0.43); sweetened milk products (0.38); cheese (0.38); sugar (0.38); sweetened beverages and energy drinks (0.37); juices (0.37) and white meat (0.34). The third dietary pattern was based on frequent consumption of sweetened milk products (0.62); breakfast cereals (0.50); milk; fermented milk drinks, and curd cheese (0.36); sweets and snacks (0.36); and infrequent consumption of egg and egg dishes (−0.37) and vegetable oils (−0.33) and was labelled ‘dairy, breakfast cereals, and treats’ (Figure 2).

### 3.3. Dietary Patterns and a Family History of P-CAD

The percentage of patients with family history of P-CAD decreased across tertiles of ‘prudent’ DP scores (test for trend *p* = 0.02), while an increase across tertiles of ‘westernized traditional’ DP scores (*p* = 0.0007) and ‘dairy, breakfast cereals, and treats’ DP scores (*p* = 0.02) was observed. Table 3.

In adjusted models, subjects with family history of P-CAD showed higher adherence by 72% to the ‘westernized traditional’ DP score in the upper tertile (OR 1.72, 95% CI (1.16–2.57), *p* = 0.007) when compared to the bottom tertile, and higher adherence by 25% per 1 unit of SD of dietary pattern score to this DP (OR 1.25, 95% CI (1.06–1.48), *p* = 0.007). Subjects with family history of P-CAD showed higher adherence by 76% or 75% in the middle or the upper tertile of ‘dairy, breakfast cereals, and treats’ DP (OR 1.76, 95% CI (1.20–2.61), *p* = 0.004 or 1.75, 95% CI (1.19–2.58), *p* = 0.005, respectively) when compared to the bottom tertile. Table 3. 

In the crude models, subjects with family history of P-CAD showed lower adherence by 18% per 1 unit of SD of dietary pattern score to the ‘prudent’ DP (OR 0.82, 95% CI (0.70–0.95), *p* = 0.01) and higher adherence by 31% per 1 unit of SD of dietary pattern score to the ‘westernized traditional’ DP (OR 1.31, 95% CI (1.12–1.53), *p* = 0.0007). No significant associations per 1 unit of SD of dietary pattern score were observed in terms of ’dairy, breakfast cereals, and treats’. In the adjusted model, family history of P-CAD remained to be associated only with ’westernized traditional’ DP (OR_adj_ 1.25, 95% CI 1.06–1.48; *p* = 0.007; per 1 unit of SD of dietary pattern score). A borderline association per one unit of SD of dietary pattern score were observed for ‘prudent’ and ‘dairy, breakfast cereals, and treats’ DPs and family history of P-CAD (*p* = 0.07 for both patterns). Table 3. 

## 4. Discussion

A family history of P-CAD is often perceived as a hereditary burden as well as a non-modifiable risk factor. The results of our study revealed for the first time that family history of P-CAD was associated with unhealthy dietary patterns, a modifiable risk factor. Young healthy adults with a family history of P-CAD were more likely to adhere to the ‘westernized traditional’ dietary pattern, in comparison to subjects without the positive family history.

The ‘westernized traditional’ and ‘prudent’ DPs are consistent with previously reported two major patterns reported in cardiovascular-related reports. The ‘westernized traditional’ pattern reflects the diet of many Poles who combine traditional staple foods (e.g., meat, potatoes, cheese) with western influences (e.g., refined grains, sweets and snacks, sweetened milk products, sugar, sweetened beverages, and energy drinks) [27,28]. The results of the nationwide project (WOBASZ), indicated that the diet of only 15% of Polish people complies with the Healthy Diet Index (HDI), and 60% presents unfavorable dietary habits (i.e., excess consumption of foods containing saturated fatty acids, and insufficient intake of foods high in folates, calcium, potassium, and magnesium) [28]. The components of the ‘westernized traditional’ pattern coincide with the characteristics reported in previous studies, in which the ‘western’ pattern was associated with an increased risk of CAD [29,30,31] and increased concentrations of CRP, insulin, C-peptide, leptin, and homocysteine [32]. Also, an increasing trend of concentration of E-selectin, sICAM-1 and sVCAM-1 across the quintiles of the ‘western’ pattern was reported, suggesting that the pathogenic mechanisms may origin in endothelial dysfunction [33]. With the global westernization, this pattern is increasingly more commonly observed in children and has been linked to increased concentrations of LDL cholesterol, triglycerides, systolic blood pressure, and fasting glucose level as early as primary and secondary school age [27,34]. The profile of this diet can be therefore interpreted as pro-atherogenic and in the long-term perspective promoting the development of coronary artery disease. Therefore, the higher adherence to this dietary pattern among young adults with family history of P-CAD is a novel but also concerning finding, that warrants further investigation. 

One of the possible explanations why the offspring of P-CAD patients were more likely to adhere to the ‘westernized traditional’ pattern is parental modelling, and the continuity of acquired habits to adult life. Parents’ behavior influences child’s dietary choices and was shown to have a lasting effect, even after the transition into independent living [35,36]. As shown by Dickens and Ogden [35] parental intake of unhealthy snacks and emotional eating predicted similar habit/behavior in young adults once they left home. Moreover, dietary patterns established in childhood and adolescence proved some consistencies over time, increasing the risk of diet-related diseases in later life [36,37]. Our results indicate that adult children of P-CAD patients may have inherited not only potential genetic susceptibility to the disease but also lifelong dietary habits, which combined with the influences of western lifestyle, may have a detrimental effect to their health, potentially accelerating disease onset. 

In our study, the crude models revealed that subjects with family history of P-CAD were less likely to adhere to the ‘prudent’ DP (by 18% per 1 unit of the dietary pattern score), in comparison to those subjects without a family history. Previous studies discovered, that adherence to dietary patterns, often labelled as ‘prudent’, ‘mediterranean’, or ‘healthy’, characterized by frequent intake of fruit, vegetables, whole grains, dairy, fish and healthy fats, have a well-documented protective effect on both, healthy individuals, and those at increased risk [28,31,32,33,34,38,39]. Interestingly, some studies reported that this effect is more pronounced in women than in men [40]. Perhaps, sex-influenced traits [40] or environmental factors, more common in men (e.g., smoking, alcohol drinking) [10], attenuated the strength of this relationship. This effect was partly reflected in our study. The observed association lost its significance after the adjustment for the confounders. Although P-CAD has a strong familial genetic component, the onset of the disease is thought to be multifactorial. Especially at the early stage—when atherosclerotic changes begin to occur—the pathogenic process can be modulated by environmental factors, including nutrition [41]. Therefore, establishing healthy dietary habits (as early in life as possible) appears to be a useful preventative measure to modulate the onset or severity of the disease. The effectiveness of the family-based approach that includes screening, lifestyle interventions, and professional support in CAD affected families is currently being investigated (PROLIFIC Study) [6]. 

The profile of ‘dairy, breakfast cereals, and treats’ DP is somewhat unique, with only few studies reporting similar results. A pattern labelled “Sweet & Dairy” (added sugar, cakes, ice-cream, coffee, eggs, butter, milk and cheese) was previously described in reports from the Italian arm of the EPIC (European Prospective Investigation into Cancer and Nutrition) study [42,43]. Also, Nobbs et al. discovered a pattern characterized by frequent consumption of breads and cereals, sweet bakery goods, and milk products [44]. However, both studies were conducted in elderly cohorts and patterns have shown only partial similarity the one described in this paper, which makes the comparison challenging. In our study, individuals with family history of P-CAD presented higher adherence to both middle and upper tertile of ‘dairy, breakfast cereals, and treats’ pattern in both, crude and adjusted model. However, the odds ratios of adherence to this pattern calculated per 1 unit of the dietary pattern score did not differ between subjects with and without a family history of P-CAD. It can be explained by no linear association between this pattern and family history. Further research could evaluate the associations between the adherence to this pattern and biomarkers of CAD in general population of young adults, regardless of their family history status. 

The main limitation of this study is that data regarding dietary habits of the parents diagnosed with P-CAD were not recorded. This information would allow confirming whether unhealthy dietary habits observed in the offspring were acquired at home and sustained over time or developed in young adulthood. However, when the study was being conducted, parents affected by P-CAD were already after a first cardiovascular event. Data collected at that time would not reflect their habitual diet prior to the onset of the disease. As research shows, a diagnosis is often a trigger for dietary and lifestyle changes [45]. Secondly, the cases had less favorable financial situation, were older and more often smokers than the controls. However, these factors were addressed in the adjusted model, where they served as confounding factors. Higher prevalence of smokers among cases can create a concern as to whether cases were less health-orientated than controls. Nonetheless, previous studies showed that patients with P-CAD are more often smokers and have less favorable dietary patterns, than patients without P-CAD [6,9,11,29,30,31]. Therefore, higher prevalence of smokers among cases may be caused by parental modelling and parenting behavior effects on offspring, rather than control group selection [35,36,37,46]. The strengths include a novel approach in investigating the associations between hereditary and environmental risks factors, and taking into account entire diet (food-based dietary patterns), rather than particular nutrients. This holistic approach allows us to form food-based recommendations fundamental to health education programs and prevention of diet-related diseases. Explained variation of dietary patterns (31%) was more than satisfactory, considering that the extend found in previous studies ranged between 13% to 30% [47]. Other assets of the study include a well-matched control group, homogenous offspring group, and verification of family history with angiographic documentation rather than self-reported data. 

## 5. Conclusions

Young healthy adults with family history of P-CAD had less favorable dietary patterns than those with negative family history. The unhealthy habits might have been established while living in the family home and tracked into the adult life, increasing already-existing familial cardiovascular risks. Primary care interventions should focus on stressing the importance of healthy diet among adults with potential hereditary susceptibility, as well as educating CAD patients about the influence of their own diet on their offspring health status.

## Figures and Tables

**Table 1:** Food groups used in dietary patterns analysis

Food Group	Questionnaire Item(s)	Remarks
Sugar	Sugar	Sugar added to beverages, such as tea, coffee, etc.
Honey	Honey	Honey added to dishes and added to beverages
Sweets and snacks	Bakers’ confectionery	Biscuits, cream cakes, sponge cakes, cheesecakes, doughnuts, poppy-seed cakes, croissants etc.
	Ice creams and custard	Ice creams and custard
	Chocolates	Chocolate, chocolate sweets and chocolate bars
	Sugar confectionery	Boiled sweets, hard caramels, jellied sweets, fudge, etc.
	Savoury snacks	Crisps, crackers, pretzels
Milk, fermented milk drinks, and curd cheese	Milk and milk beverages—natural	Milk and natural milk beverages (yoghurt, kefir, buttermilk), porridge, etc.
	Cheese curds	Cheese curd, natural cottage cheese, soft cheese, mozzarella, cottage cheese with herbs, etc.
Sweetened milk products	Milk beverages—sweetened	Fruit yoghurts, yoghurts with chocolate flakes, flavoured buttermilk, hot chocolate, etc.
	Flavoured cheese curds	Flavored curds (with fruit, chocolate, vanilla), etc.
Cheeses	Cheese	Hard cheese, blue cheese, processed cheese, cheese spreads, etc.
Eggs and egg dishes	Eggs and egg dishes	Scrambled eggs, omelette, egg salad, cooked eggs
Breakfast cereals	Breakfast cereals	Muesli, cornflakes, other cereals—sweetened or unsweetened, etc.
Wholegrain products	Wholemeal cereals	Wholemeal wheat or rye bread, seeded loafs, pumpernickel, wholemeal cracker bread, etc.
	Coarse groats	Buckwheat groats, barley, brown rice, wholemeal pasta, etc.
Refined grain products	Refined cereals	White bread, rye, wheat-rye bread, toast bread, white bread rolls, brioche, bagels, etc.
	Fine groats	Semolina, milled barley, pasta, white rice, rice flakes, etc.
Animal fats	Butter	Butter
	Cream	Single, double, sour, used as an ingredient or added to beverages
	Other animal fats	Lard, pork fat, etc.
Red meats	Red meat	Pork, beef, veal, etc.
Venison	Venison	Wild boar, venison, quail, mallard, hare, etc.
Processed meats	Sausages, bacon, reconstituted meat	Sausages, meat loaf, hot-dogs, smoked sausages, bacon, etc.
	High quality cured meats	Ham, poultry and pork-beef good quality cold meats, etc.
	Offal products	Liver, blood sausage, sweetbread, liver pate, etc.
Vegetables	All kind of vegetables (cruciferous, root, yellow-orange, leafy green, tomatoes, gourds, and squashes)	Cabbages, Brussel sprouts, cauliflower, broccoli, kale, carrots, peppers, spinach, chicory, lettuce, rocket, leek, celery, parsley, tomatoes, fresh cucumber, marrow, courgettes, pumpkins, aubergines, Parsnip, beetroots, onion, garlic, celeriac, radishes, turnip, salads, mixed vegetables
Potatoes	Potatoes	Boiled, baked, French fries, potato rosti, gnocchi, etc.
Vegetable oils	Vegetable based oil	
Other edible fats	Margarine	Margarine for baking, frying, spreading
	Mayonnaise	Mayonnaise and salad dressings
White meat	Poultry and rabbit	
Fish	Lean fish	Pollock, cod, perch, hake, carp to 1 kg, tuna, panga, trout etc.
	Oily fish	Salmon, sardines, herring, mackerel, eel, large carp etc.
Fruit	All kind of fruits (stone fruits, kiwi and citrus fruits, tropical fruits, berries, bananas, apples, and pears)	Apricots, cherries, nectarines, peaches, plums, grapes, kiwi, oranges, mandarins, grapefruit, lemons, pomelos, pineapples, watermelon, melons, fresh dates and figs, strawberries, raspberries, blackberries, blueberries, redcurrants, blackcurrants, bananas, apples, pears
Nuts and seeds	Nuts and nut spreads	Peanuts, hazelnuts, walnuts, cashews, coconuts, chestnuts, etc.
	Seeds and bran	Pumpkin seeds, sesame seeds, sunflower seeds, wheat germs, wheat bran, etc.
Legumes	Fresh and tinned legumes	Corn, green peas, green beans, etc.
	Dry and processed pulses	Beans (fava, butter kidney, broad, French, green), soya, peas, chickpea, and processed pulses (baked beans, hummus, other bread spreads)
Juices	Fruit juices and nectars	Mixed fruit juice, orange, grapefruit, apple, pear, grape, blackcurrant, cherry juice
	Vegetable and vegetable-fruit juices	Mixed vegetable juice, tomato, carrot and carrot-fruit juice
Sweetened beverages and energy drinks	Sweetened beverages	
	Energy drinks	
Alcohol	Beer	Beer
	Wine and cocktails	Wine and cocktails
	Spirits	Vodka and other spirits

**Table 2:** Characteristics of the study sample.

Variable	Total Sample	Family History of P-CAD		*p*
		With	Without	
*N* (%)	689 (100.0)	351 (50.9)	338 (49.1)	
Age (years)	28.0 (SD 4.5)	28.6 (SD 4.6)	27.3 (SD 4.2)	0.0001
Female	299 (43.4)	142 (40.5)	157 (46.4)	0.11
Current smoking (vs. past smoker or non-smoker)	154 (22.4)	96 (27.4)	58(17.2)	0.001
Place of residence—city >20,000 inhabitants (vs. city <20,000)	514 (74.6)	252 (71.8)	262 (77.5)	0.08
Financial situation above average (vs. average or below average)	173 (25.1)	74 (21.1)	99 (29.2)	0.01
Higher education (vs. secondary or primary)	389 (56.4)	195 (55.6)	194 (57.4)	0.63
Physical activity at leisure time				
Low	171 (24.8)	90 (25.6)	81 (24.0)	0.78
Moderate	328 (47.6)	168 (47.9)	160 (47.3)	
High	190 (27.6)	93 (26.5)	97 (28.7)	

Values are mean and standard deviation (SD) or *N* (%).

**Table 3:** Sample percentage (%), crude and adjusted odds ratios (ORs (95% CI)) of a family history of P-CAD, according to dietary patterns (*n* = 689).

Dietary Patterns	Family History of P-CAD								
	With		Without	With					
	%	*p* for Trend	OR	OR_crude_	95% CI	*p*	OR_adj_ *	95% CI	*p*
‘Prudent’									
Bottom tertile (ref.)	56.1	0.02	1.0 (ref.)	1.0 (ref.)	-----------	----	1.0 (ref.)	--------	-------
Middle tertile	51.3		1.0	0.82	0.57–1.19	0.30	0.94	0.64–1.39	0.75
Upper tertile	45.4		1.0	0.65	0.45–0.94	0.02	0.72	0.48–1.09	0.11
Per 1 unit of SD of dietary pattern score	-----	------	1.0	0.82	0.70–0.95	0.01	0.85	0.72–1.01	0.07
‘Westernized traditional’									
Bottom tertile (ref.)	43.5	0.0007	1.0	1.0 (ref.)	-----------	-------	1.0 (ref.)	------------	-------
Middle tertile	50.0		1.0	1.30	0.90–1.88	0.16	1.24	0.85–1.82	0.26
Upper tertile	59.4		1.0	1.90	1.31–2.76	0.0007	1.72	1.16–2.57	0.007
Per 1 unit of SD of dietary pattern score	-----	------	1.0	1.31	1.12–1.53	0.0007	1.25	1.06–1.48	0.007
‘Dairy, breakfast cereals, and treats’									
Bottom tertile (ref.)	44.3	0.02	1.0	1.0 (ref.)	------------	--------	1.0 (ref.)	-----------	-----
Middle tertile	53.5		1.0	1.44	1.00–2.09	0.05	1.76	1.20–2.61	0.004
Upper tertile	55.0		1.0	1.54	1.06–2.21	0.02	1.75	1.19–2.58	0.005
Per 1 unit of SD of dietary pattern score	------	------	1.0	1.11	0.95–1.29	0.19	1.16	0.99–1.36	0.07

* OR_adj_—odds ratio adjusted for age (years), sex, smoking status (never-smoker or former smoker, current smoker), place of residence (city <20,000 inhabitants, city >20,000 inhabitants), financial situation (below average or average, above average), education (primary or secondary, higher), physical activity at leisure time (low, moderate, high).
